# Cocoa Farmers’ Compliance with Safety Precautions in Spraying Agrochemicals and Use of Personal Protective Equipment (PPE) in Cameroon

**DOI:** 10.3390/ijerph15020327

**Published:** 2018-02-13

**Authors:** Abayomi Samuel Oyekale

**Affiliations:** Department of Agricultural Economics and Extension, North-West University, Mafikeng Campus, Mmabatho 2735, South Africa; asoyekale@gmail.com

**Keywords:** agrochemical, cocoa farmers, safety compliance, personal protective equipment, Cameroon

## Abstract

The inability of farmers to comply with essential precautions in the course of spraying agrochemicals remains a policy dilemma, especially in developing countries. The objectives of this paper were to assess compliance of cocoa farmers with agrochemical safety measures, analyse the factors explaining involvement of cocoa farmers in the practice of reusing agrochemical containers and wearing of personal protective equipment (PPE). Data were collected with structured questionnaires from 667 cocoa farmers from the Centre and South West regions in Cameroon. Data analyses were carried out with Probit regression and Negative Binomial regression models. The results showed that average cocoa farm sizes were 3.55 ha and 2.82 ha in South West and Centre regions, respectively, and 89.80% and 42.64% complied with manufacturers’ instructions in the use of insecticides. Eating or drinking while spraying insecticides and fungicides was reported by 4.20% and 5.10% of all farmers in the two regions, respectively. However, 37.78% and 57.57% of all farmers wore hand gloves and safety boots while spraying insecticides in the South West and Centre regions of Cameroon, respectively. In addition, 7.80% of all the farmers would wash agrochemical containers and use them at home, while 42.43% would wash and use them on their farms. Probit regression results showed that probability of reusing agrochemical containers was significantly influenced (*p* < 0.05) by region of residence of cocoa farmers, gender, possession of formal education and farming as primary occupation. The Negative Binomial regression results showed that the log of number PPE worn was significantly influenced (*p* < 0.10) by region, marital status, attainment of formal education, good health, awareness of manufacturers’ instructions, land area and contact index. It was among others concluded that efforts to train farmers on the need to be familiar with manufacturers’ instructions and use PPE would enhance their safety in the course of spraying agrochemicals.

## 1. Introduction

Cocoa cultivation in Cameroon is primarily embraced by peasant farmers, who may own one to three hectare cocoa farms [1]. Out of the four regions where cocoa is grown, the South West region is the highest producer, accounting for about 60 percent of Cameroon’s total annual output [2]. Recent growth in international demand for cocoa has caught the government’s attention, with the Cameroonian government setting a target of increasing the output to 600,000 tonnes by 2020. If achieved and all things being equal, Cameroon would become the third largest producer of cocoa in the world [3]. However, some expert opinions have highlighted the inadequate supply of necessary farm inputs and growing incidences of cocoa pests and diseases as major obstacles to achieve such a significant increase in cocoa output. Currently, as the fifth world’s largest producer of cocoa, Cameroon realizes about £308.6 million annually from international cocoa trades, which also constitutes about 50% of the country’s total revenues from primary agricultural exports [2,3].

Moreover, cocoa production in Cameroon is facing significant setbacks from unfavourable government policies, climatic change, pest infestations, disease outbreaks and inefficiency of the different marketing outlets. Specifically, the major pests affecting cocoa production in Cameroon are myrid bugs, most importantly *Sahlbergella singularis*, while *Phytophthora megakarya* is largely responsible for black pod disease [4]. The use of agrochemicals by cocoa farmers is therefore indispensable in order to minimize losses of potential outputs as a result of pests and diseases. Similarly, as soil fertility reduces, the need to apply fertilizers on cocoa trees becomes imperative [5]. International Labour Organization (ILO) [6] reported that in Cameroon, farmers were using agrochemicals such as fungicides (Ridomil, Nordox), insecticides (Gamaline, Cypercal), herbicides and fertilizer. The study showed that some thirty five different agrochemicals comprising four herbicides, eleven fungicides and twenty insecticides were marketed for use on Cameroon’s cocoa farms.

The fundamental concern of agricultural policy makers is that in some instances agrochemicals that had been banned in developed countries could still be freely sold for some domestic and agricultural uses in some developing countries [7]. This goes in line with the findings of International Labour Organization (ILO) [6] that in Cameroon, there were at least eight active agrochemical ingredients which were officially banned but still being used by cocoa farmers. Similarly, it was reported that more than 77% of the farmers did not follow basic recommendations on dosage and safety procedures while applying agrochemicals on their farms. 

There is the need to exercise extra care while applying agrochemicals to crops due to adverse health hazards they could constitute [8]. Some agrochemicals have been linked to deficiency in the functioning of the human immune systems [9,10,11]. The compounded impacts of such exposure would culminate into health problems such as asthma, low sperm count and sterility [12,13]. In addition, it had been emphasized that exposure to agrochemicals could be linked to diseases such as cancer, Alzheimer’s disease, type 2 diabetes, Parkinson’s disease, endocrine disruption, learning and cognitive development disorders and birth defects, among others [14]. Individuals who are regularly exposed to agrochemicals have a very high risk of being diagnosed with pancreatic cancer [15,16], prostate cancer [17,18] and myeloid leukaemia [19,20,21]. Besides some other risk factors, breast cancer diagnoses have been linked to exposure to agrochemicals [22,23]. 

Accordingly, the prescriptive emphases of agrochemical producers on the need to ensure adequate protection for people spraying them are meant to guarantee safety and reduce their associated occupational health hazards. Compliance with safety instructions on the use of agrochemicals and minimization of their associated health hazards are therefore paramount issues for which policy interventions are still needed in many African cocoa producing countries. In Cameroon, ILO [6] reported that 64% of sampled cocoa farmers did not adhere to recommended doses of agrochemicals. In 2015, Cameroon approved more than 600 agrochemicals for use with insecticides, herbicidedes and fungicides constituting the highest percentages with 33.93%, 26.55% and 24.26%, respectively. Similarly, based on some international classification codes, 65% of these agrochemicals would require to be sprayed after being mixed with water. Therefore, with cases of accidental poisoning from agrochemicals becoming significant public health problem in Cameroon, institutional lapses in providing some coordinative roles through some form of toxicovigilance system has been highlighted [24]. It had also been noted that there is complete absence of any form of control from designated government agencies and departments to monitor and reduce misuse of agrochemicals and facilitate farmers’ compliance with some basic safety measures [25].

Between 2001 and 2002, report from a pilot survey which was conducted in Cameroon indicated that while low quality of spraying equipment was reported to have contributed negligibly to agrochemical accidents among farmers, absence of PPE played significant role. It was also noted that farmers were not complying with the requirement of disposing agrochemical properly with more than 60% constituting some environmental health risks through the practice of pouring agrochemical leftovers into nearby rivers or streams [26]. Many previous studies in Cameroon have highlighted inadequate use of PPE and the risk of water contamination resulting from agrochemicals usage by farmers [26,27,28,29,30]. In some similar studies, Tarla et al. [31] found that manufacturers’ instructions on agrochemicals were not followed by more than 76% of Cameroonian farmers, while only 2.1% reported to have wore PPE. Among some sampled vegetable farmers in Cameroon, it was reported that a majority of the farmers overused some agrochemicals [32]. Such overdose usage constitutes a health hazard for numerous households in Cameroon due to presence of agrochemical residues in some food products [33].

In some other studies, Bassi et al. [34] noted that ignorance on the part of some Nigerian farmers about the health hazards that are associated with agrochemicals often induces their usage at high dosages. Huang et al. [35] and Xu [36] noted that illiterate farmers face challenges in comprehending written instructions on agrochemicals and cannot access other useful information unless it is imparted verbally or through some practical demonstration. Therefore, wearing of personal protective equipment (PPE) may not be taken seriously by farmers who are illiterate. Perry et al. [37] found that compliance with wearing of protective gear during application of dicamba, atrazine, and cyanazine was very low among some field crop farmers. 

Schenker et al. [38] found that in California, being young and being a male were positively associated with wearing of PPE. However, no significant relationship was found with cigarette smoking, farm residence and marital status. Bosompem and Mensah [39] found that among selected cocoa farmers in Ghana, wearing of hand gloves, masks and protective clothes (coats) in the course of applying agrochemicals was very low with 35.6%, 37.5% and 35.4% usage, respectively. Wearing of Wellington boots had the highest usage, with approximately 66%. Olowogbon [40] also found that about 35% of sampled farmers in Nigeria were wearing PPE. Okoffo et al. [41] found that 35% of cocoa farmers in selected villages in Ghana were wearing full PPE comprised of a protective hat, hand gloves, goggles to protect the eyes, nose masks to prevent inhalation, overalls and rubber boots, while 45% worn them partially. The study found that the factors that explained decision to wear PPE were educational levels of farmers, years of experience in cocoa farming, farm size, farmers’ age, agricultural extension contacts and presence of agrochemical shops in the villages.

This paper seeks to add to existing literature on cocoa farmers’ compliance with safety measures in the course of spraying agrochemicals. The specific objectives are to describe the behaviour of cocoa farmers while spraying agrochemicals, analyse the factors explaining involvement of cocoa farmers in the practice of reusing agrochemical containers and analyse the factors explaining wearing of personal protective equipment (PPE) while spraying agrochemicals. It was hypothesized that decision to reuse agrochemical containers by cocoa farmers has nothing to do with their socio-economic characteristics and the amount of PPE worn by cocoa farmers while spraying agrochemicals is not in any way related to their socio-economic characteristics. This study will contribute to policy debates on food safety and occupational health hazards in Cameroon.

## 2. Materials and Methods

### 2.1. The Study Areas

**Figure 1:**
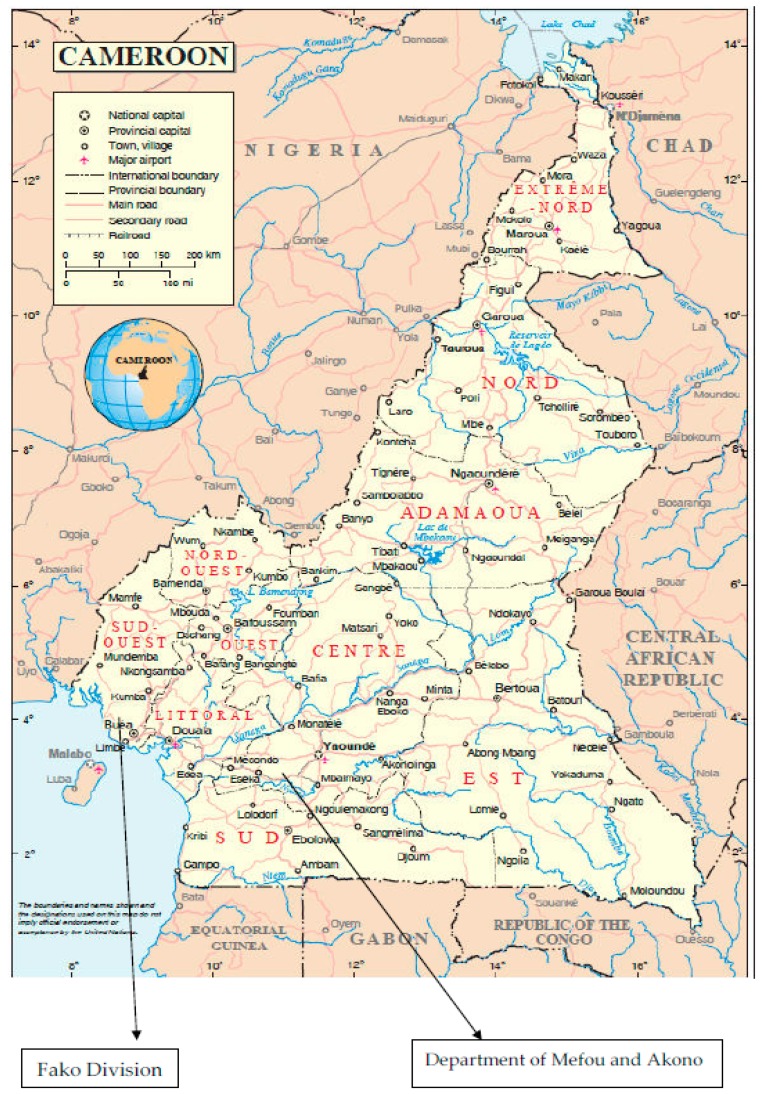
Map of Cameroon showing cocoa growing regions and division where data were collected (Source: http://valliscommodities.com/wp-content/uploads/2015/04/May-2012_Cameroon-Cocoa-Industry.pdf).

The data for this study were collected from Fako division in the South West region and Department of Mefou and Akono in the Centre region of Cameroon in March 2015 and December 2014, respectively. Buea is the most notable town in Fako division, while Ngoumou is the capital of Department of Mefou and Akono. Fako division lies to the West of Yaounde and its capital (Buea) is 60 km from Douala [42]. The region is at an altitude of 1000 m above sea level with a favourable climate for cocoa agriculture. However, Ngoumou is located about 45 km from Yaounde. It is spread over an area of 400 km square with only 100 km^2^ inhabited. The remaining 300 km^2^ are still occupied by cultivable forests, where about 56 villages are located and agricultural activities are predominantly subsistence [43]. Figure 1 shows these two divisions within the cocoa growing regions in Cameroon.

### 2.2. Sampling Methods

Twenty enumerators were engaged for data collection. These comprised 10 students each from the University of Yaounde II and the University of Buea. The enumerators from the University of Yaounde II interviewed cocoa farmers in Ngoumou (the Department of Mefou and Akono) while those from the University of Buea were trained to interview cocoa farmers in Fako division. The training was to ensure proper understanding of the contextual interpretations of the questions in the questionnaire and to highlight some ethical standards to be adhered with during data collection. The training section lasted for about three hours at each of the venues.

The questionnaire was subjected to face validity by seeking comments from some agricultural research experts. Comments that were received during face validation led to modification, removal and general re-editing of some questions. It should also be noted that having been administered previously among cocoa farmers in Ghana, this questionnaire had gone through extensive academic scrutiny that enhanced its validity. The training sections that were organized for the enumerators also generated some questions, which brought further clarity to some initially designed questions. Some questionnaires were pretested on few cocoa farmers in selected villages before they were administered to intended respondents. The purpose of pretesting was to validate the questions and detect any form of ambiguity.

The respondents were sampled using a multi-stage sampling procedure. The first stage involved random selection of villages from the list provided by the coordinating agricultural extension officers who acted as local guides. Thereafter, households were randomly sampled based on population proportional to size. The total number of respondents from Fako division was 402 from 27 villages, while 265 cocoa farming households from 22 villages were sampled from the Department of Mefou and Akono. Questionnaires for farmers in Department of Mefou and Akonowere written in French, while those for farmers from Fako division were written in English. This is a reflection of the bilingual status of Cameroon as a country where English and French speakers coexist.

The interview was conducted with one farmer at a time by enumerators who guided the interviewees in the whole processes of data collection. The, interview, which lasted for about between 20–30 min had none of the farmers refusing to be interviewed because the local guides properly informed their community leaders about the study. Similarly, no form of incentive was given to the farmers and they all showed willingness to participate without being forced. The questionnaire, which was tagged “Agrochemical safeguard measures and health of cocoa farmers” had four sections. Section A was on farmers’ demographic information, section B was on safeguard measures that are always taken in the course of using agrochemicals, section C is on agrochemical exposure risk and health problems being suffered by farmers and section D was on farmers’ stress and occupational hazards on cocoa farms. These are some of the questions contained in the questionnaire: Are you aware that there are some safety measures to be taken while applying these agrochemicals on your crop? Do you follow manufacturers’ instructions before using the following agrochemicals? If you must carry chemical sprayers on your back, are you always assisted by someone? When spraying the following agrochemicals on cocoa farms, do you spray along the wind direction? Information on the types of agrochemical that were being used by the farmers was not collected because the study focused on compliance with agrochemical safety measures given the general understanding of their highly corrosive and poisonous properties.

### 2.3. Estimated Models

#### 2.3.1. Probit Regression Model

One of the major instructions on agrochemicals is that the containers must be properly disposed with strict warning of never to attempt reusing them for any purpose. However, some cocoa farmers are in the habit of converting agrochemical containers for some farm and domestic uses. This increases their risk of injecting these poisonous agrochemicals into their body systems. In order to determine the factors explaining such exposure, cocoa farmers’ decision to reuse agrochemical containers was modeled with Probit regression. The dependent variable, which captures exposure risk factor is binary in nature with users of agrochemical containers coded 1 and 0 otherwise. This type of data cannot be modeled with Ordinary Least Square (OLS) regression as a result of significant violation of some of its basic assumptions. Following Cappellari and Jenkin [44], the estimated model is expressed as:

(1) $$Y_{i}=\alpha +\overset{k}{\underset{i=1}{\sum }}\beta_{k}X_{k}+u_{i}$$

where *Y_i_* represents a binary dependent variable coded as 1 if farmers were reusing agrochemical containers and 0 otherwise. $X_{k}$ is a vector of independent variables and $u_{i}$ represents the stochastic error term. α and β are the estimated parameters while *k* denotes the number of explanatory variables.The model’s explanatory variables are presented in Table 1. Multicollinearity among the variables was addressed by running the model using OLS and invoking the *vif* command in STATA13 software (StataCorp LLC, College Station, TX, USA). Multicollinearity is considered as a serious problem when the variance inflation factor (VIF) is 10 and above [45,46]. The marginal parameters were computed by invoking *mfx* command after running the Probit regression.

#### 2.3.2. Negative Binomial Regression Model

Negative Binomial regression model was used to analyze the determinants of the number of personal protective equipment (PPE) that farmers wear while spraying agrochemicals. In this case, the dependent variable is a count, which is an integer. Following Zwilling [47] and Hilbe [48], the estimated model can be specified as follows:

(2) $$ln\;\mu_{i}=\rho +\overset{k}{\underset{i=1}{\sum }}\pi_{k}X_{k}+v_{i}$$

where $ln\;\mu_{i}$ is the natural log of the number of PPE worn while spraying different agrochemicals (insecticides, herbicides, fungicides and fertilizers). $X_{k}$ is a vector of independent variables already described in Table 1 and $v_{i}$ represents the stochastic error term. ρ and π are the estimated parameters, while *k* denotes the number of explanatory variables in the model. It should be noted that Negative Binomial regression was used in this study after Poisson regression failed to properly fit the data. This was confirmed by invoking the *estat gof* command in the STATA 13 software in order to compute the Pearson goodness of fit and Deviance goodness of fit statistics after estimating the Poisson regression model. The conclusion that Poisson regression was inadequate was reached based on statistical significance (*p* < 0.05) of computed Pearson and Deviance goodness of fit statistics.

## 3. Results

### 3.1. Demographic Characteristics of Sampled Cocoa Farmers

Table 2 presents the demographic characteristics of the respondents across the selected regions in Cameroon. It shows that average age for the combined data from the two regions was approximately 47 years. South West farmers were significantly (*p* < 0.01) younger, with an average age of 45.20 years compared to an average of 50.09 years for those from Centre region. The results further showed that 71% and 70% of the farmers in the Centre and South West regions were married, respectively, while 12% and 5% were separated or divorced. Male farmers constituted 91% of the cocoa farmers in each of the regions. Attainment of formal education was very high at 97% for the combined farmers. There is no significant difference between average years of education in the Centre (9.46) and South West (9.22) regions (*p* > 0.05), while average years of education was 9.3 in the combined data. In addition, 87% of all the farmers indicated that farming was their primary occupation. 

Table 2 further shows that average household sizes in Centre and South West Cameroon were significantly different (*p* < 0.05) with 7.04 and 6.46, respectively. Average farming experience was also significantly higher (*p* < 0.01) in the Centre region with 28.50 years, as compared to 16.22 in the South West region. Also, farmers from the Centre region had significantly higher (*p* < 0.05) average years of growing cocoa (22.25), as compared with those from the South West region with 13.72. In addition, the peasant nature of cocoa agriculture in Cameroon is well reflected in average cocoa farm sizes which are 2.82 ha and 3.55 ha in the Centre and South West regions respectively and being statistically different (*p* < 0.01).

### 3.2. Agrochemical Exposure Risk Factors among Cocoa Farmers

#### 3.2.1. Activities during Spraying of Agrochemicals

Table 3 contains data on activities of cocoa farmers while spraying agrochemicals. It reveals that 89.80% of the farmers from South West were following manufacturers’ instructions in the use of insecticides, versus 42.64% for the Centre region. Also, 82.09% of the farmers from South West region followed manufacturers’ instructions on the use of herbicides. In the combined data, 81.26% followed manufacturers’ instructions on the use of fungicides. The results in Table 3 further show that majority of the farmers did not have anyone to assist them in lifting agrochemical sprayers and properly fixing them on their backs. Specifically, in the course of using insecticides, 7.92% and 8.71% of the farmers were assisted in lifting sprayers to their backs in the Centre and South West regions, respectively. Also, 13.21% and 7.71% indicated being assisted in lifting fungicides to their backs in Centre and South West regions, respectively.

The results also indicate that higher number of farmers from South West region were spraying along wind directions. In the combined data, 61.11% and 66.87% indicated that they normally spray insecticides and fungicides, respectively along wind directions. Some farmers indicated that they do eat or drink while spraying agrochemicals. Specifically, from Table 3, 4.20% and 5.10% of all the sampled farmers would eat or drink in the course of applying insecticides and fungicides, respectively.

#### 3.2.2. Wearing of Personal Protective Equipment (PPE)

Wearing of PPE is essential in order to reduce human contacts with agrochemicals. The results in Table 4 show that 37.78% of all the farmers indicated that they normally wore hand gloves in the course of spraying insecticides. This is quite lower than the proportion that were wearing safety boots (57.57%). Fewer than one-quarter of the farmers wore protective clothes and goggles while spraying insecticides, herbicides and fertilizers. It should also be noted that compliance with wearing of PPE was generally higher among farmers from South West region, although majority of them were not wearing protective clothes.

#### 3.2.3. Methods of Disposing Agrochemical Containers

Another vital risk factor for spraying agrochemicals is the method of disposing of the containers. The expectation of manufacturers is that after usage, the containers would be properly disposed of, preferably by burying them. The results in Table 5 show that 7.80% of all the farmers would wash the containers and reuse them at home. In some instances, such uses include serving as storage for palm oil, salt and other food spices. Similarly, 42.43% of all the farmers would wash and reuse agrochemical containers on their farms. In South West region, 17.41% of the farmers indicated that they do wash and reuse agrochemical containers on their farms as, against 80.38% in the Centre region. In some instances, such farm uses could be as storage for seeds, salt, palm oil and other food spices. They may also serve as containers for fetching and drinking water. Table 5 also reveals that 37.31% of the farmers in South West region indicated that they always bury the containers as against 5.28% in the Centre region. However, 34.08% and 4.15% of the farmers from South West and Centre regions, respectively indicated that they would throw the containers anywhere on their farms.

Table 6 presents what farmers were doing with unused mixed agrochemicals. Such leftovers are expected to be disposed of safely inside a dug hole (distant away from rivers or streams) right in the midst of the farm. However, the results indicate that 7.95% and 8.85% of the farmers were disposing leftovers of insecticides and fungicides inside some dug holes on the farm. In South West region, majority of the farmers would retain such leftovers of mixed agrochemicals for use at another time, while some poured them into running streams or rivers.

### 3.3. Factors Explaining Decision to Reuse Agrochemical Containers

Table 7 presents the Probit regression results. It shows that the model produced a good fit based on statistical significance of the Likelihood Ratio Chi Square (*p* < 0.01). This implies that estimated parameters are not jointly equal to zero. The first null hypothesis is hereby rejected. 

Multicollinearity was not a problem given the low value (1.6) of the computed variance inflation factor (VIF). The variables that showed statistical significance (*p* < 0.05) were region, male farmers (gender), formal education and farming as a primary occupation. These results indicate that farmers from the South West region had significantly lower probability of reusing agrochemical containers. Specifically, from the marginal estimated parameters, the probability of reusing agrochemical’s containers reduced by 0.5535 as a result of residence in South West region, other factors being held constant. Also, the coefficient of gender implies that male farmers had higher probability of reusing agrochemical containers. The marginal coefficient shows that being a male cocoa farmer increased the probability of reusing agrochemical containers by 0.1971. The coefficient of attainment of formal education is negatively signed and implies that farmers with formal education had lower probability of reusing agrochemical containers. It should be noted that household size coefficient is with positive sign but shows no statistical significance (*p* > 0.05).

### 3.4. Factors Explaining Wearing of PPE

Table 8 presents the results of factors influencing the number of personal protective equipment that farmers wore in the course of spraying agrochemicals. Multicollinearity was also not a problem given the low value of the computed VIF of 1.60. The computed Likelihood Ratio Chi Square statistics is statistical significance (*p* < 0.01) in all the estimated models. This implies that estimated parameters were not jointly equal to zero (0) in all the models, and hypothesis two should be rejected.

The coefficients of region in all the estimated models have a positive sign and are statistically significant (*p* < 0.01). These results imply that compared to farmers from the Centre region, those from South West region wore more PPE worn when spraying insecticides, herbicides and fertilizers. The coefficient of being married shows statistical significance (*p* < 0.05) in the insecticide model. The result indicates that compared to those who were single, farmers that were married, the amount of PPE worn when applying insecticides was higher by an average of 0.2251. The coefficient of separated/divorced has a negative sign and is statistically significant (*p* < 0.05) in the model for herbicides. The results indicated that compared to those farmers who were single, the log of the number of PPE worn when spraying herbicides by farmers that were separated/divorced reduced by an average of 0.7553. The coefficients of good health are all with positive sign and statistically significant (*p* < 0.01) in all the estimated models. These imply that compared to those without good health, the log of the number of PPE worn by healthy farmers increased by average of 0.4626, 0.5226, 0.3732 and 0.5338 in the course of spraying insecticides, herbicides, fungicides and fertilizers, respectively. Awareness of manufacturers’ instruction is with positive parameters and statistically significant (*p* < 0.01) for models estimated for insecticides, herbicides and fertilizers. These results imply that farmers that indicated that compared those farmers that were not aware, being awareness of manufacturers’ instructions increased the log of the number of PPE worn by average of 2.0608, 1.2807 and 1.0204 when spraying insecticides, herbicides and fertilizers, respectively. 

Land area variable is having a coefficient that is statistically significant (*p* < 0.05) in the estimated model for insecticides. This implies that as cocoa land areas increases by one hectare, farmers’ log of number of PPE worn increases by 0.0191. Also, the coefficients of agrochemical contact index are with negative sign in all the estimated models and statistically significant (*p* < 0.01). These imply that as agrochemical contact index increases by one unit, log of number of PPE worn decreased by 0.0942, 0.0490, 0.0826 and 0.0730 when spraying insecticides, herbicides, fungicides and fertilizers, respectively.

## 4. Discussion

Cocoa farmers in Cameroon were found to be ageing. This is worth noting because one of the major issues in promoting output growth in Cameroon’s cocoa agriculture is ageing of cocoa farmers [3]. Ageing of farmers had been linked to low cocoa productivity in some cocoa growing African countries [49]. Previously, it was found that average age of some sampled cocoa farmers in Cameroon was 52 years [1], while average age of cocoa farmers in South West Cameroon was 40.3 years [50]. As expected, cocoa farming is also dominated by men. This goes in line with previous findings [1,50]. Several other studies had emphasized male dominance in cocoa agriculture. This could be traced to tediousness of cocoa farming which traditionally ascribes its operations to men [39,51,52]. Cameroonian cocoa farmers were moderately educated. Previous study [50] submitted that average year of education among cocoa farmers in South West Cameroon was 7.56. The agrarian nature of the study areas is reflected by dominance of farming as primary occupation. The results also indicate involvement of more youths in cocoa production in South Western region of Cameroon. Low average farm sizes among cocoa farmers is an issue which had been previously reported [53]. This may also contribute to low socio-economic status of cocoa farmers due to inability to benefit from some economies of scale advantages.

Many cocoa farmers from the Centre region were not following manufacturers’ instructions during usage of agrochemicals. This could be more of an attitude problem. The consciousness of toxicity nature of agrochemicals often decreases after being used over time. This implies that if contact with an agrochemical is not causing any immediate discomfort such as itchy skin, sneezing or coughing, farmers may consider them to be less toxicy. Therefore, when farmers lack clear knowledge of the cumulative impacts of agrochemicals, they may not be able to ensure maximum protection for themselves while applying them on their farms.

More importantly, Damalas and Eleftherohorinos [54] noted that farmers’ exposure to agrochemicals increases when keen attention is not paid to manufacturers’ instructions. Ignorance on the precautions to be taken for storage, spraying and recommended dosage often subjects many expose users of agrochemicals to several health risks and the often severe consequences of misapplication [55,56]. The South Australia Environmental Protection Agency [57] submitted that it is important for users of agrochemicals to understand the instructions that are guiding their usage in order to minimize associated hazards of misapplication. Damalas and Eleftherohorinos [54] also noted that the form in which agrochemicals are formulated can determine the level of exposure by end users. Specifically, there are tendencies for liquid agrochemicals to splash during the course of usage, thereby contacting users’ skin and other parts of the body. Similarly, agrochemicals that are in powder form can be blown into the air, thus facilitating their being inhaled or coming into contact with farmers’ eyes. 

Some farmers reported inability to seek assistance in lifting knapsack sprayer containers, especially when it is filled to the brim with mixed liquid agrochemicals. This poses some risk of exposure to many of the farmers. This becomes more critical when the containers are leaking or not properly covered. In case of spraying agrochemicals in a windy environment, proper understanding of the wind direction would minimize unintentional or accidental contacts with farmers [57]. This issue is most applicable to insecticides and fungicides which would have to be sprayed on cocoa pods from a certain distance. 

The practice of eating while spraying agrochemicals was also reported among cocoa farmers. This is quite risky because it increases the likelihood of direct oral injection of agrochemicals. Damalas and Eleftherohorinos [54] noted that exposure of farm workers to agrochemicals increases when the basic recommendation of properly washing hands after spraying or before eating is not observed. Majority of the cocoa farmers were also wearing their normal farm clothes while spraying agrochemicals. This may not prevent penetration of liquid agrochemicals from the reach of the skin in case of any accidental spills or unexpected change of wind directions. 

Non-compliance of cocoa farmers with wearing of PPE poses significant threats to their health. In a study in Ghana, Okoffo et al. [41] found that only 20% of the farmers ignored the need to wear PPE while spraying agrochemicals. The study by Bakhsh et al. [58] also showed low compliance with PPE usage among cotton farmers in Pakistan. In another study among vegetable farmers in Nigeria, Ugwu et al. [59] found that in the course of spraying agrochemicals, 95.3% of the farmers claimed to be using rubber gloves, 83.3% used nose guards, 83.3% used protective clothes, 62.7% worn caps and 60.0% put on face masks. 

Furthermore, high usage of safety boots among cocoa farmers was reported. This is a reflection of their multi-purpose use. Farmers work in highly hazardous environment with high risk of stepping on thorns, sharp stumps, snakes, scorpions and other unforeseeable hazard components on the farms. Wearing of safety boots gives some form of protection to farmers in the event of accidental contacts with any of such highlighted occupational health hazard sources. Similar findings had been reported by Damalas and Abdollahzadeh [60]. In a study by Andrade-Rivas and Rother [61], it was noted that farm workers who are engaged in spraying herbicides did not perceive these chemicals as an important health concern when gauged with some other occupational health hazards. It was noted that the decision to use PPE could be informed by the speed of completing an assignment and associated comfort. Prioritization of other farm hazards from thorns promotes wearing of safety boots, while the less familiar health risk of being exposed to herbicides reduces wearing of other PPE. Similar practices had been reported by Halfacre-Hitchcock et al. [62] among some migrant farm workers in the United States of America where hand gloves were worn more to ensure protection from some job-related discomfort rather than for preventing contacts with pesticides.

The results re-emphasized involvement of cocoa farmers in some risk-prone behaviours of reusing agrochemical containers. This behaviour contravenes the standard practice for handling toxic substances. The toxicity property of agrochemicals seems not to be deeply understood by some cocoa farmers. This is because farmers could think that if washed properly, containers of agrochemical could be safe for domestic and farm uses. That is why some would take the pain of washing them with hot water and soap. Whichever way the washing could have been done, reuse of agrochemical containers is very dangerous. In another study, Yang et al. [63] found that majority of the farmers in the Wei River catchment of China discarded empty containers of pesticides near their farms, while less than 20% disposed of the containers along with other refuse, burnt or buried them. In this study, some farmers were also pouring agrochemical leftovers inside some nearby running rivers or streams. Depending on the concentration, aquatic organisms could be affected, while downstream residents along the water course may ignorantly drink polluted and contaminated water. Also, some cocoa farmers were merely pouring leftovers of agrochemicals anywhere on their farms. This can constitute environmental and health risks if erosion carries the agrochemical residues into nearby streams or rivers. 

Moreover, the results indicated lesser involvement in the behaviour of reusing agrochemical containers by farmers from the South West region. Also, farmers from the South West region were more compliant with PPE usage than their counterparts in the Centre region. The South West region is the highest producer of cocoa in Cameroon. Also, there is concentration of interventions for promoting sustainable cocoa production in this region with significant awareness on proper ways of spraying agrochemicals. High usage of PPE could be a reflection of high cocoa productivity in South West Cameroon, which could facilitate investment in activities that would minimize occupational health hazards of the farmers. 

Attainment of formal education also reduced reuse of agrochemical containers. This is in line with an a priori expectation because educated farmers would be able to read and understand instructions on use of agrochemicals, which often emphasize the risk of reusing their containers. One of the major issues is ignorance of the properties of some chemical compounds that agrochemicals are made of. Some farmers believe that once washed thoroughly with water and soap, containers of agrochemicals would be for some domestic and farm uses. Attainment of formal education may also increase farmers’ awareness of the long term health consequences of being exposed to agrochemicals, thereby inducing compliance with behaviours that would facilitate protection from any form of contact. More importantly, educated farmers may possess the requisite knowledge of the carcinogenic properties of some chemical compounds that are found in agrochemicals. Education could also reduce misapplication and misuse of agrochemicals.

Cocoa land areas increased the number of PPE worn while spraying insecticides. This is expected because farmers with large cocoa farms would require a lot of days to complete spraying of insectides and the need to guarantee less contacts with the chemicals would be critical. This finding could also reflect ability of cocoa farmers with large farmlands to realize enough income from cocoa production that would facilitate their investment in PPE. 

Being married increased the amount of PPE worn while spraying insecticides. This reflects some notion of responsible behaviour expected from family men. The perception of cocoa farmers about their being in good health status increased the use of PPE. This is expected because compliance with safety instructions in respect of spraying agrochemicals by wearing PPE could reduce health hazards of farmers and make them relatively healthier that those not complying. Awareness of manufacturers’ instruction also enhanced wearing of PPE. This finding is in line with expectation since farmers who are aware of manufacturers’ instructions are more likely to take some precautions while spraying agrochemicals. Result from contact indices are also in line with expectation given that contacts with agrochemicals will be predominant among those farmers that are not complying with safety requirements. 

## 5. Conclusions

This paper analysed the behaviours of cocoa farmers that could promote their exposure to toxic agrochemicals and determined the factors explaining reuse of agrochemical containers and wearing of PPE. One of the major limitations of this study was the inability to observe each of the cocoa farmers individually on their farms while spraying agrochemicals. This was informed by limited financial and time resources that were available to implement the surveys. When such direct observation is possible, it may inform some key findings which may not have been necessarily captured in the questionnaire on behavioural attitudes and actions of farmers while using agrochemicals. Failure to include qualitative research design that uses Focused Group Discussions (FGDs) constitutes another limitation. This would be able to unfold some hidden issues underlying non-compliance with basic safety measures while spraying agrochemicals.

Ensuring cocoa farmers’ compliance with safety requirements in spraying agrochemicals is essential in order to reduce persistent health and environmental hazards that are associated with such negligence. The case for Cameroon requires urgent attention given rapid changes in morbidity and health complaints by the population. Possession of knowledge on the health hazards and environmental impacts of misuse and misspraying of agrochemicals would enhance the need to safeguard workers that are directly involved in spraying these toxic chemicals by ensuring some protective behaviour.

It was found that cocoa farmers were involved in some risky behaviours such as non-compliance with wearing of PPE, improper disposal of agrochemical containers like retention for domestic and farm uses and disposal of unused leftovers of agrochemicals inside rivers and anywhere on the farm. It was also found that regional variable, awareness of manufacturers’ instructions, marital status, being healthy and exposure index significantly influenced the number of PPE worn, while involvement in risky behaviour of reusing agrochemical containers increased among male farmers, those from the Centre region, illiterate farmers and those farming on part-time basis.

There are several environmental and health implications that could be drawn from this study. First, reuse of agrochemical containers, eating while spraying agrochemicals and pouring of agrochemical leftovers into water bodies increases the risk of exposure to chemical compounds. Low usage of PPE also exposes farmers to the risk of being exposed to agrochemicals. This poses some serious health concerns as a result of the toxicity properties of some chemical compounds that these agrochemicals contain.

The findings also point at the need for the proper creation of awareness among different farmers’ groups and training of cocoa farmers on essential safety precautions which are to be observed in the course of spraying agrochemicals. Such training could be implemented by agricultural extension agents. Based on the findings, the bulk of these problems is among farmers from the Centre region, male farmers and those with no formal education. Utilization of the farmers’ groups would enhance proper coverage under limited.

However, the Farmers’ Field School (FFS) as a platform for providing informal education to farmers could integrate such training into their programmes with some practical demonstrations. In addition, several other media such as radio and television could be explored to educate farmers on the consequences of non-compliance with agrochemical usage instructions. Such programmes should also create awareness on essential agrochemical guidelines for users and educate farmers on those agrochemicals that are prohibited from being used in Cameroon. Farmers need to understand the cumulative health impacts of agrochemicals which requires their being properly protected at all times. They should also be dissuaded from believing that once washed properly, agrochemical containers are safe for domestic and farm uses.

## Figures and Tables

**Table 1:** Description of explanatory variables that were used in the analyses.

Variables Descriptions	Coding Method
Region	South West = 1, 0 otherwise
Age of farmers	Years
Marital status (married)	Yes = 1, 0 otherwise
Marital status (separated/divorced)	Yes = 1, 0 otherwise
Gender (male farmer)	Yes = 1, 0 otherwise
Has formal education	Yes = 1, 0 otherwise
Farming as primary occupation	Yes = 1, 0 otherwise
Household size	Number of persons
Farmers in good health	Yes = 1, 0 otherwise
Awareness of agrochemical usage instructions	Yes = 1, 0 otherwise
Land areas	Hectares
Agrochemicals contacts index—generated with Principal Component Analysis (PCA) using farmers responses to whether they inhale, swallow or have other contacts with agrochemicals during spraying.	Continuous values

**Table 2:** Descriptive statistics of farmers’ demographic characteristics.

Region	Centre		South West		All Farmers		
Demographic Characteristics	Mean	Std Dev.	Mean	Std Dev.	Mean	Std Dev.	*t*-test Statistics
Age of farmer	50.09	12.340	45.20	13.619	47.15	13.333	−4.800 ***
Married (yes = 1, 0 otherwise)	0.71	0.457	0.70	0.459	0.70	0.458	-
Separated/Divorced (yes = 1, 0 otherwise)	0.12	0.326	0.05	0.223	0.08	0.271	-
Male (yes = 1, 0 otherwise)	0.91	0.282	0.91	0.286	0.91	0.284	-
Formally educated (yes = 1, 0 otherwise)	0.98	0.149	0.96	0.201	0.97	0.183	-
Years of education	9.46	4.307	9.22	4.788	9.31	4.602	−0.980
Farming as primary occupation (yes = 1, 0 otherwise)	0.88	0.322	0.85	0.354	0.87	0.342	-
Household size	7.04	3.962	6.46	3.380	6.69	3.631	−1.967 **
Farming experience (years)	28.50	15.639	16.22	11.906	21.10	14.780	−10.868 ***
Cocoa farming experience (years)	22.25	15.752	13.72	10.232	17.11	13.372	−7.791 ***
Farm size (hectares)	2.82	2.09	3.55	3.79	3.28	3.24	3.235 ***

*** denotes statistically significant at 1%; ** denotes statistically significant at 5%.

**Table 3:** Percentage distribution of cocoa farmers’ based on safety related activities in the course of handling agrochemicals.

Activities	Follow Manufacturers’ Instructions			Someone Helped to Fix Sprayers at the Back			Spray along Wind Direction			Eat or Drink during Spraying		
Region/Agrochemical	Centre	South West	All	Centre	South West	All	Centre	South West	All	Centre	South West	All
Insecticide	42.64	89.80	71.06	7.92	8.71	8.40	39.02	75.62	61.11	5.66	3.23	4.20
Herbicide	6.06	82.09	51.95	1.13	6.72	4.50	2.64	69.40	42.88	0.00	2.24	1.35
Fertilizer	5.28	78.11	49.18	0.38	7.21	4.50	2.64	63.18	39.13	4.15	2.24	3.00
Fungicide	76.98	84.08	81.26	13.21	7.71	9.90	60.00	71.39	66.87	9.06	2.49	5.10

**Table 4:** Percentage distribution of cocoa farmers based on wearing of safety kits during handling of agrochemicals.

	Hand Glove	Safety Boots	Protective Clothes	Goggle	Ventilation Mask
Centre Region	%	%	%	%	%
Insecticide	17.74	27.92	13.21	12.08	14.34
Herbicide	1.89	3.40	1.13	0.75	1.89
Fertilizer	1.51	2.64	1.51	1.13	1.51
Fungicide	33.58	53.96	27.17	25.28	29.06
South West Region					
Insecticide	51.00	77.11	32.34	31.59	37.31
Herbicide	45.02	68.16	30.50	26.37	31.84
Fertilizer	45.77	65.67	28.36	25.87	32.59
Fungicide	46.27	69.90	29.60	27.86	34.58
All Farmers					
Insecticide	37.78	57.57	24.74	23.84	28.19
Herbicide	27.89	42.43	18.80	16.19	19.94
Fertilizer	28.19	40.63	17.69	16.04	20.24
Fungicide	41.23	63.57	28.64	26.84	32.38

**Table 5:** Percentage distribution of cocoa farmers based on methods of disposing containers of agrochemicals.

Group	South West Region	Centre Region	All Farmers
Wash and Use at Home	8.21	7.17	7.80
Wash and Use on Farm	17.41	80.38	42.43
Bury it	37.31	5.28	24.59
Throw it on farm	34.08	4.15	22.19
Others	2.99	3.02	2.99

**Table 6:** Percentage distribution of cocoa farmers based on ways of disposing left over of unused mixed agrochemicals.

	Retain for Use Next Time	Pour in a Stream	Pour on Cocoa Farm	Pour near Cocoa Trees	Pour inside Hole Dug	Pour inside a Container
Centre Region	%	%	%	%	%	%
Insecticides	27.17	1.89	4.91	3.77	8.68	5.28
Herbicides	3.40	0.00	0.75	0.38	0.76	0.00
Fertilizers	3.41	0.00	0.75	0.38	0.75	1.51
Fungicides	40.75	5.66	11.70	7.55	13.21	7.17
South West Region						
Insecticides	74.38	4.23	5.97	8.21	7.46	5.47
Herbicides	68.41	4.48	4.48	4.73	5.22	5.22
Fertilizers	64.43	4.98	4.23	6.97	6.22	5.74
Fungicides	69.65	4.48	5.24	5.72	5.97	4.98
All Farmers						
Insecticides	55.62	3.30	5.55	6.45	7.95	5.40
Herbicides	42.58	2.70	3.00	3.00	3.45	3.15
Fertilizers	40.24	3.00	2.85	4.35	4.05	4.05
Fungicides	58.17	4.95	7.81	6.45	8.85	5.85

**Table 7:** Probit regression results of factors explaining reuse of agrochemical containers by cocoa farmers.

	Standard Probit			Marginal Effect		
Explanatory Variables	Coefficient	Std. Err.	*z*-Stat	dy/dx	Std. Err.	*z*-Stat
Region	−1.559046 ***	0.2137972	−7.29	−0.55354 ***	0.05996	−9.23
Age of farmer	0.0053399	0.0047443	1.13	0.0021269	0.00189	1.13
Married	−0.1809712	0.1455006	−1.24	−0.0717852	0.05737	−1.25
Separated/divorce	0.180683	0.2653679	0.68	0.0713527	0.10345	0.69
Male farmer	0.5050666 **	0.2189954	2.31	0.1971413 **	0.08108	2.43
Formal education	−0.8796388 ***	0.3137156	−2.80	−0.3070321 ***	0.08484	−3.62
Farming as primary occupation	−0.4770359 ***	0.1695095	−2.81	−0.1836394 ***	0.06155	−2.98
Household size	0.0326645	0.0172165	1.90	0.0130103	0.00686	1.90
Good health	−0.0121746	0.1666117	−0.07	−0.004848	0.06633	−0.07
Awareness of agrochemical	−0.268433	0.1870791	−1.43	−0.106436	0.07361	−1.45
Land area	−0.021997	0.0202979	−1.08	−0.0087614	0.00809	−1.08
Exposure index	−0.0122127	0.0271864	−0.45	−0.0048643	0.01083	−0.45
Constant	1.679394 ***	0.4725411	3.55			
Log likelihood function	−313.028					
Likelihood Ratio Chi Square	293.04 ***					
Pseudo R square	0.3188					

Note: *** denotes statistically significant at 1%; ** statistically significant at 5%.

**Table 8:** Negative Binomial regression results of factors explaining number of personal protective equipment (PPE) worn.

	Use PPE for Insecticides		Use of PPE for Herbicide		Use of PPE for Fungicides		Use of PPE for Fertilizer	
Explanatory Variables	Coefficient	*z*-Stat	Coefficient	*z*-Stat	Coefficient	*z*-Stat	Coefficient	*z*-Stat
Region	2.3616 ***	7.85	2.1360 ***	7.92	0.2688	1.75	2.5088 ***	9.14
Age of farmer	0.0018	0.62	0.0040	1.06	0.0051	1.68	0.0041	1.06
Married	0.2251 **	2.50	0.1294	1.14	0.1659	1.73	0.1998	1.69
Separated/divorced	−0.1318	−0.70	−0.7553 ***	−2.75	−0.3279	−1.81	−0.1185	−0.48
Male farmer	−0.2080	−1.76	−0.1963	−1.23	−0.0089	−0.07	−0.0625	−0.37
Formal education	0.0659	0.36	0.2499	1.01	0.3095	1.41	0.5492	1.91
Farming as primary occupation	−0.1349	−1.42	−0.0286	−0.22	−0.0052	−0.05	−0.0994	−0.75
Household size	0.0030	0.28	−0.0018	−0.13	−0.0053	−0.5	0.0185	1.29
Good health	0.4626 ***	3.31	0.5226 ***	3.01	0.3732 ***	3.4	0.5338 ***	3.04
Awareness of agrochemical instructions	2.0608 ***	7.95	1.2807 ***	5.92	0.1353	0.95	1.0204 ***	5.01
Land area	0.0191 **	2.42	0.0136	1.14	0.0039	0.35	0.0162	1.33
Agrochemical contact index	−0.0942 ***	−6.2	−0.0490 **	−2.56	−0.0826 ***	−4.41	−0.0730 ***	−3.66
Constant	−3.9471 ***	−9.89	−3.3906 ***	−7.85	−0.5130	−1.6	−4.0886 ***	−8.78
Ln alpha	-3.8511		−1.1481		−1.0712		−1.0360	
Alpha	0.0213		0.3172		0.3426		0.3549	
Log likelihood	−752.891		−805.235		−1203.42		−800.064	
LR Chi Square (12)	621.11 ***		421.10 ***		453.02 ***		406.61 ***	
Likelihood-ratio	0.29		42.74 ***		71.29 ***		41.42 ***	

Note: *** denotes statistically significant at 1%; ** statistically significant at 5%.
